# Patient experiences with the role of physical activity in inflammatory bowel disease: results from a survey and interviews

**DOI:** 10.1186/s12876-021-01739-z

**Published:** 2021-04-14

**Authors:** Carlijn R. Lamers, Nicole M. de Roos, Lola J. M. Koppelman, Maria T. E. Hopman, Ben J. M. Witteman

**Affiliations:** 1 Department of Gastroenterology and Hepatology, Hospital Gelderse Vallei, Willy Brandtlaan 10, 6716 RP Ede, The Netherlands; 2 Division of Human Nutrition and Health, Wageningen University and Research (WUR), Stippeneng 4, 6708 WE Wageningen, The Netherlands; 3 Department of Physiology, Radboud University Medical Center, Philips Van Leydenlaan 15, 6500 HB Nijmegen, The Netherlands

**Keywords:** Crohn’s disease, Ulcerative colitis, Physical activity, Interviews

## Abstract

**Background:**

Physical activity may affect disease activity in patients with inflammatory bowel disease. We used a survey to investigate this association and performed interviews to get a better understanding of patient experiences, and therefore the nature of this association.

**Methods:**

Patients with Crohn’s disease (CD, n = 176) and ulcerative colitis (UC, n = 162) completed the short Crohn’s Disease Activity (sCDAI) or Patient Simple Clinical Colitis Activity Index (P-SCCAI) and the Short Questionnaire to Assess Health-enhancing physical activity (SQUASH). Associations were investigated by multiple linear regression. Semi-structured interviews (7 CD, 7 UC) were conducted to assess patient experiences with the role of physical activity in their disease.

**Results:**

The majority of survey participants were in remission (70%) and adhered to the Dutch physical activity guidelines (61%). In Crohn’s disease, the total physical activity score was inversely associated with disease activity, even after adjustment for confounders (β = − 0.375; *p* = 0.013). No association between physical activity and disease activity was found in ulcerative colitis. Of the interviewees, 86% experienced beneficial effects of physical activity, such as improved general fitness, quality of life and self-image. However, during periods of active disease they struggled to find the motivation and perseverance to be physically active due to physical barriers.

**Conclusions:**

Crohn’s disease participants with a higher physical activity level had a lower disease activity. This inverse association was not found in ulcerative colitis. Interviews revealed that IBD patients generally experience beneficial effects from physical activity, although the barriers caused by active disease may put them off to be physically active.

**Supplementary Information:**

The online version contains supplementary material available at 10.1186/s12876-021-01739-z.

## Background

Physical activity might have a protective role in the development of Crohn’s disease (CD) and ulcerative colitis (UC), and possibly also supports maintenance of remission and improves quality of life in patients with one of these inflammatory bowel diseases (IBD) [1]. An increasing number of studies suggest that low to moderate intensity exercise might be beneficial for IBD patients by increasing health-related quality of life and reducing inflammation [2]. Although the exact mechanism is unknown, modification of the gut microbiota and influence of physical activity on immunological processes have been proposed as possible routes of action [3, 4]. Another way in which physical activity might be beneficial, is by reducing psychological stress. In a longitudinal study in sixty UC patients in remission, researchers found that stressful events were associated with higher chances of relapse [5]. Moreover, some studies suggest that physical activity might reduce fatigue levels in patients with IBD [6, 7].

Despite the potential beneficial effects of exercise, a recent cross-sectional study showed that patients were significantly less physically active after their IBD diagnosis than before. It is not clear whether this was due to discomforts from their disease or to a certain fear that exercise would worsen their disease progression or symptoms [8]. In surveys, patients report to experience barriers to exercise due to conditions related to their IBD such as fatigue, joint pain and weakness, and fear for symptom exacerbation [9]. They also report complaints during physical activity such as an increased urgency and abdominal pain, making it hard to complete the exercise [9, 10]. However, interviews explicitly addressing patient experiences are lacking.

Since IBD patients experience less complaints during remission than during active disease, it is vital to sustain remission as long as possible [11]. Although medication is the predominant form of treatment [12], physical activity could be a complementary therapy that can be implemented easily in a patient’s daily routine. Consequently, it is valuable to understand the relation between physical activity and disease activity in IBD patients, and how this affects well-being. Therefore, the aim of this study was to assess the association between physical activity and disease activity in a large group of IBD patients including both CD and UC patients, and to explain the nature of this association by interviews.

## Methods

### Study design and study population

This study consisted of an online survey and interviews. The online survey was part of a larger survey about lifestyle factors and disease activity [13], which was conducted between July and October 2018. The survey was composed of questions regarding disease activity, physical activity and participants’ characteristics. IBD patients aged 18 years or older, diagnosed with either CD or UC, were included. Details about recruitment can be found elsewhere [13]. In total, 397 patients showed interest in the study and received access to our online survey. We excluded participants with indeterminate colitis, unknown IBD type or incomplete physical activity data.

For the interviews, participants who had not taken part in the survey were recruited between December 2019 and July 2020 via the outpatient clinic of Hospital Gelderse Vallei, Ede, the Netherlands. The same in- and exclusion criteria as applied for the survey were used. The interviews focussed on effects of physical activity on disease activity, fatigue and quality of life, and vice versa. In total, fourteen interviews were conducted. This sample size was chosen because data saturation is typically reached between ten to fifteen semi-structured interviews [14, 15], which also applied to this study.

The medical ethical committee of Wageningen University decided that no formal ethical approval was needed, due to the low burden and risk of the study. All participants provided digital or written informed consent.

### Data collection

#### Survey

The short Crohn’s Disease Activity Index (sCDAI) and the Patient Simple Clinical Colitis Activity Index (P-SCCAI) were used to assess disease activity [16, 17]. Disease activity was used as a continuous outcome measure and classified into remission, mildly, moderately and severely active disease using previously validated cut-off points [16, 18].

Physical activity was assessed using the validated Short Questionnaire to Assess Health-enhancing physical activity (SQUASH). The SQUASH contains questions regarding multiple activities during an average week in the past month, namely commuting activities, leisure time activities, household activities and activities at work or school. The number of days per week, average time per day and intensity of every activity was reported [19]. The total physical activity score was calculated by summing up the different activity scores which were calculated by the number of minutes per week of that activity times the corresponding metabolic equivalent of task (MET) [20]. The total physical activity score was used to investigate the association between physical activity and disease activity.

Besides, information was collected on age, gender, height and weight, level of education, type of IBD, age at diagnosis, current medication, previous IBD-related surgeries and smoking.

#### Interviews

Interviews were explorative and semi-structured according to an interview guide (Additional file 1), and were all performed by the same researcher. The interviews mainly consisted of open-ended questions about IBD-related complaints, physical activity in general, the link between physical activity and IBD, fatigue, and quality of life. These questions could lead to follow-up questions, which were impromptu. This semi-structured method is suitable for interpretation and exploration of wishes, attitudes, perceptions, and opinions of interviewees [21]. Nine interviews were held face-to-face and five interviews were conducted via telephone due to COVID-19. All interviews were recorded after which they were transcribed verbatim. Recordings were removed immediately after full transcription and were not shared with persons outside the research team. During transcription, names were coded to ensure anonymity. All interviews were conducted in Dutch. Quotes originating from the interviews were translated into English.

### Data analysis

#### Survey

Data are presented as mean ± standard deviation (SD) for normally distributed continuous data or median with interquartile range (IQR) when skewed. Categorical data are presented as counts and percentages. To test for differences between CD and UC and between disease activity groups, independent samples t-test and one-way analysis of variance (ANOVA) were performed for continuous variables, or Kruskall-Wallis test when not normally distributed. Post-hoc analyses for disease activity groups were performed using the Bonferroni multiple comparisons test. For categorical data, Chi-square tests were performed. Multiple linear regression was used to determine associations between total physical activity score and disease activity, and was adjusted for age, gender, BMI and education level in the first model, and also for age at diagnosis, medication use and previous IBD-related surgery in the second model. Results were reported as β-coefficients (per 100 point change in total physical activity score) with 95% confidence intervals (95% CI). A *p* value of < 0.05 was considered statistically significant. Statistical analysis was carried out using IBM SPSS Statistics version 24.

#### Interviews

Analysis of the transcripts was done by inductive coding with Atlas.ti 8. In vivo codes and codes assigned by the researcher were used to code the data. Four main themes emerged from the analysis. Within these themes, results were analysed on corresponding and contradicting answers.

## Results

### Participants’ characteristics

**Fig. 1:**
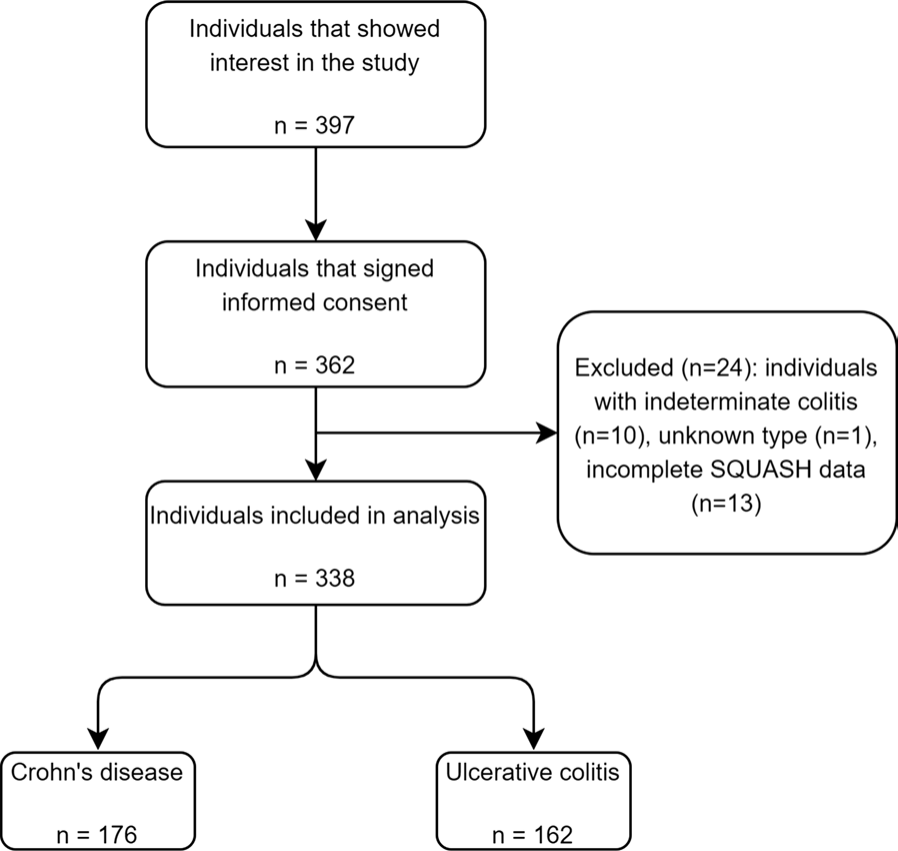
Flowchart of individuals included in analysis

For the survey, 338 participants were included in the analysis of which 176 participants (52%) had CD and 162 participants (48%) had UC (Fig. 1). About two-thirds of participants were female and close to half of participants was highly educated and had experienced no flare-ups in the last year. As only two UC participants were classified as having severely active disease, they were included in the moderately active disease group. About two-thirds of participants were in remission. CD participants were slightly younger at diagnosis, used more immunosuppressants and biologicals, and had more IBD-related surgeries than UC participants (Table 1). Interviews were conducted with fourteen IBD patients of which seven had CD and seven had UC. The interviewees were 25 to 78 years of age (median 61 years), and the number of males and females was equal. Mean body mass index (BMI) of the interviewees was 26.3 ± 7.1 kg/m^2^ and they were low (n = 4), middle (n = 5) and highly (n = 5) educated (Additional file 2: Supplementary Table S1).

**Table 1:** Characteristics of the survey study population consisting of 176 CD and 162 UC participants

	CD	UC	Disease activity CD			Disease activity UC		
			Remission	Mild	Moderate	Remission	Mild	Moderate
Subjects, n (%)	176 (52.1)	162 (47.9)	127 (72.2)	31 (17.6)	18 (10.2)	110 (67.9)	36 (22.2)	16 (9.9)
Gender, n (%)								
Female	124 (70.5)	101 (62.3)	89 (70.1)	19 (61.3)	16 (88.9)	65 (59.1)	26 (72.2)	10 (62.5)
Age (years)	47.6 ± 15.6	50.0 ± 14.5	47.8 ± 15.7	47.2 ± 16.6	46.7 ± 13.9	49.8 ± 14.0	52.5 ± 15.4	45.4 ± 15.5
Age at diagnosis (years)	32.3 ± 14.6*	35.4 ± 14.1*	32.6 ± 15.1	30.9 ± 14.8	32.6 ± 9.4	34.3 ± 13.3	39.7 ± 15.5	33.4 ± 15.5
BMI (kg/m^2^)	24.9 ± 4.7	24.9 ± 3.8	25.0 ± 4.4	24.6 ± 4.8	25.0 ± 6.6	24.6 ± 3.5	25.4 ± 3.9	25.6 ± 5.6
Smoking, n (%)								
Never	127 (72.2)	125 (77.2)	99 (78.0)^a^	19 (61.3)^a,b^	9 (50.0)^b^	83 (75.5)	28 (77.8)	14 (87.5)
Current	18 (10.2)	6 (3.7)	10 (7.9)	6 (19.4)	2 (11.1)	4 (3.6)	1 (2.8)	1 (6.3)
Former	31 (17.6)	31 (19.1)	18 (14.2)^a^	6 (19.4)^a,b^	7 (38.9)^b^	23 (20.9)	7 (19.4)	1 (6.3)
Education level^#^, n (%)								
Low	40 (22.7)	32 (19.8)	27 (21.3)	8 (25.8)	5 (27.8)	21 (19.1)	8 (22.2)	3 (18.8)
Middle	53 (30.1)	50 (30.9)	38 (29.9)	6 (19.4)	9 (50.0)	35 (31.8)	8 (22.2)	7 (43.8)
High	83 (47.2)	80 (49.4)	62 (48.8)	17 (54.8)	4 (22.2)	54 (49.1)	20 (55.6)	6 (37.5)
Medication use, n (%)								
Mesalazines	29 (16.5)**	102 (63.0)**	22 (17.3)	4 (12.9)	3 (16.7)	69 (62.7)	20 (55.6)	13 (81.3)
Corticosteroids	24 (13.6)	23 (14.2)	14 (11.0)	7 (22.6)	3 (16.7)	10 (9.1)^a^	9 (25.0)^b^	4 (25.0)^a,b^
Immunosuppressants	76 (43.2)**	33 (20.4)**	55 (43.3)	14 (45.2)	7 (38.9)	20 (18.2)	8 (22.2)	5 (31.3)
Biologicals	57 (32.4)**	28 (17.3)**	35 (27.6)^a^	12 (38.7)^a,b^	10 (55.6)^b^	11 (10.0)^a^	9 (25.0)^a,b^	8 (50.0)^b^
Other	24 (13.6)	12 (7.4)	14 (11.0)	6 (19.4)	4 (22.2)	5 (4.5)^a^	3 (8.3)^a,b^	4 (25.0)^b^
No medication use	36 (20.5)	29 (17.9)	30 (23.6)	5 (16.1)	1 (5.6)	23 (20.9)	6 (16.7)	0 (0.0)
Flare-ups in past year, n (%)								
None	91 (51.7)*	63 (38.9)*	79 (62.2)^a^	7 (22.6)^b^	5 (27.8)^b^	51 (46.4)^a^	10 (27.8)^a,b^	2 (12.5)^b^
1–2 flare-ups	59 (33.5)	68 (42.0)	38 (29.9)	15 (48.4)	6 (33.3)	42 (38.2)	21 (58.3)	5 (31.3)
3–4 flare-ups	9 (5.1)*	18 (11.1)*	2 (1.6)^a^	5 (16.1)^b^	2 (11.1)^a,b^	9 (8.2)^a^	3 (8.3)^a^	6 (37.5)^b^
More than 4 flare-ups	17 (9.7)	13 (8.0)	8 (6.3)^a^	4 (12.9)^a,b^	5 (27.8)^b^	8 (7.3)	2 (5.6)	3 (18.8)
Surgery, n (%)	60 (34.1)**	13 (8.0)**	41 (32.3)	10 (32.3)	9 (50.0)	6 (5.5)	6 (16.7)	1 (6.3)

Data are presented as mean ± SD for normally distributed data. Categorical data is presented as n (%). *p < 0.05; **p < 0.01

*CD* Crohn’s disease, *UC* ulcerative colitis, *BMI* body mass index

^#^Education level: no education, primary or lower vocational education and lower general secondary education (low); secondary vocational education and higher general secondary education (middle); higher vocational education and university (high)

^ab^Groups with the same superscript letters do not differ significantly after post-hoc analyses using the Bonferroni test (*p* > 0.05)

### Survey

**Fig. 2:**
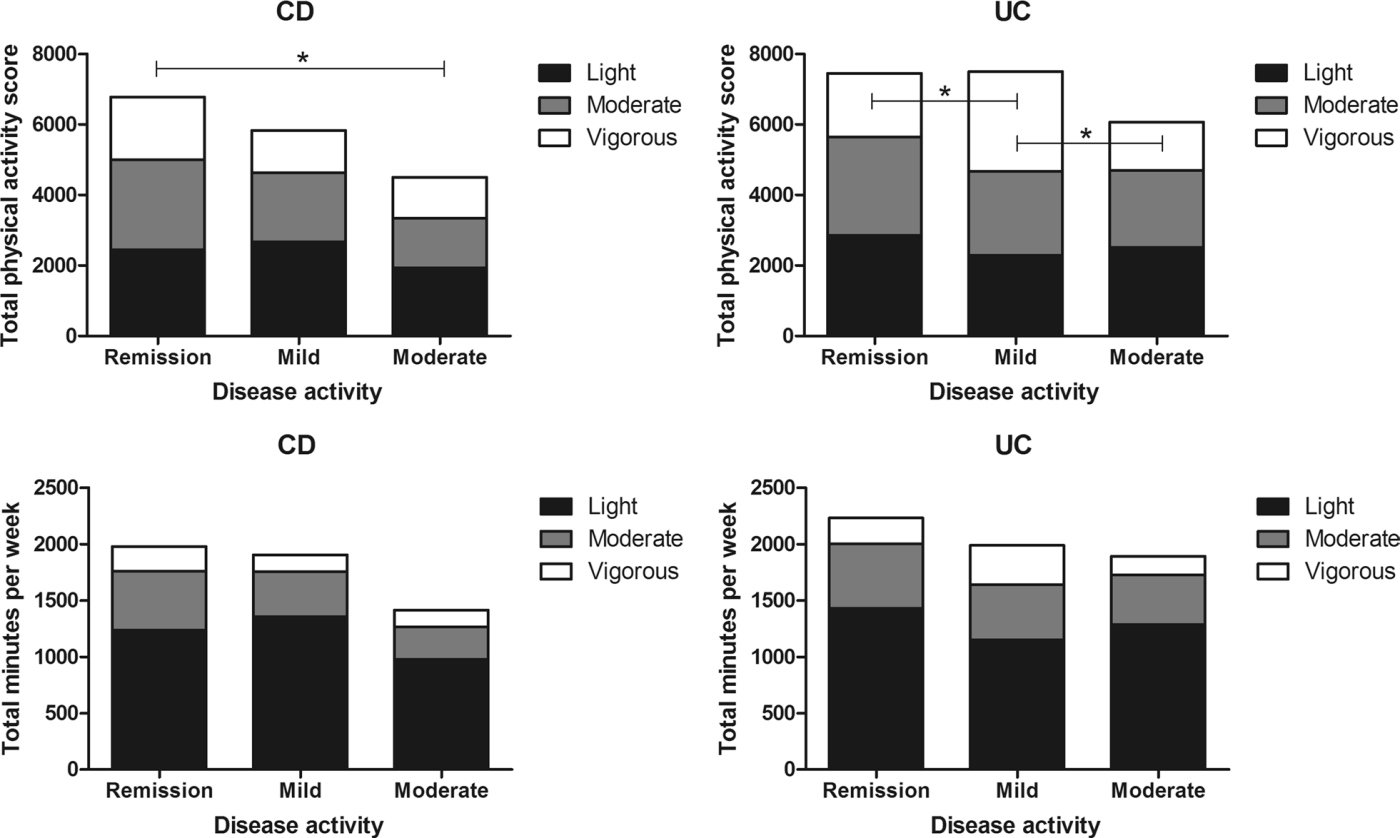
Total physical activity score (top) and total minutes per week (bottom) of CD and UC participants stratified for disease activity, divided into light, moderate and vigorous physical activity. Total physical activity score was calculated by summing up the different activity scores which were calculated by the number of minutes per week of that activity times the corresponding metabolic equivalent of task (MET). *p < 0.05. *CD* Crohn’s disease, *UC* ulcerative colitis

Significant differences were found between CD and UC regarding total physical activity scores and total minutes per week, with UC participants being more physically active than CD participants (*p* = 0.035) and performing activities with a higher intensity resulting in a higher total physical activity score (*p* = 0.025) (Table 2). In CD participants, multiple linear regression showed an inverse association between the total physical activity score and disease activity scores in the crude model (β = − 0.406; *p* = 0.007) (Table 3). This suggests that CD participants who are more physically active and probably perform activities with a higher intensity, have a lower disease activity. After adjustment for age, gender, BMI and education level, and additionally age at diagnosis, medication use and previous IBD-related surgery, this association remained (β = − 0.375; *p* = 0.013) (Table 3). No significant association was found between the total physical activity score and UC disease activity scores (β = − 0.006; *p* = 0.162) (Table 3). Across disease activity groups, the total physical activity score was significantly lower in CD when disease activity was higher (*p* = 0.035), with a significant difference between the remission and moderately active disease group after post-hoc analyses (*p* = 0.046) (Table 2, Fig. 2). When disease activity is higher, the number of minutes per week spent on physical activity is the same as for the lower disease activity groups, but participants seem to choose activities with a lower intensity (Table 2, Fig. 2).

**Table 2:** Disease activity and physical activity scores of CD and UC participants and stratified for disease activity

	CD	UC	Disease activity CD			Disease activity UC		
			Remission	Mild	Moderate	Remission	Mild	Moderate
Disease activity								
sCDAI score	95 [48–158]	^–^	79 [44–107]^a^	175 [165–198]^b^	269 [233–326]^c^	–	–	–
Range	44–357	–	44–146	150–219	220–357	–	–	–
P-SCCAI score	^–^	1 [1–3]	^–^	^–^	^–^	1 [1, 2]^a^	3 [3, 4]^b^	7 [6–9]^c^
Range	–	0–13	–	–	–	0–2	3–5	6–13
Physical activity								
Total physical activity score	6382 ± 3747*	7329 ± 3989*	6782 ± 3963^a^	5834 ± 2863^a,b^	4501 ± 2858^b^	7457 ± 4052	7501 ± 4160	6070 ± 3039
Total minutes per week	1907 ± 1031*	2143 ± 1016*	1979 ± 1071	1903 ± 871	1413 ± 895	2230 ± 1035	1989 ± 998	1890 ± 890

Data are presented as mean ± SD for normally distributed data or median [interquartile range] when skewed. *p < 0.05

*CD* Crohn’s disease, *UC* ulcerative colitis, *sCDAI* short Crohn’s Disease Activity Index, *P-SCCAI* Patient Simple Clinical Colitis Activity Index

^ab^Groups with the same superscript letters do not differ significantly after post-hoc analyses using the Bonferroni test (*p* > 0.05)

**Table 3:** Results of multiple linear regression of the association between physical activity with disease activity as continuous variables, for CD and UC

	CD (n = 176)		UC (n = 162)	
	β-coefficient (95% CI)^a^	*p* value	β-coefficient (95% CI)^a^	*p* value
Total physical activity score				
Crude	− 0.406 (− 0.698 to 0.114)	**0.007**	− 0.006 (− 0.015 to 0.003)	0.162
1	− 0.395 (− 0.693 to 0.097)	**0.010**	− 0.005 (− 0.014 to 0.004)	0.293
2	− 0.375 (− 0.668 to 0.081)	**0.013**	− 0.003 (− 0.013 to 0.006)	0.490

CD disease activity scores can range from 0 to > 450 and UC disease activity scores can range from 0 to 19

Bold values are significant

*CD* Crohn’s disease, *UC* ulcerative colitis, *CI* confidence interval

^a^Per 100 point increase in total physical activity score

Model 1: adjusted for age (years), gender (m/f), BMI (kg/m^2^) and education level (low/middle/high)

Model 2: as model 1 plus age at diagnosis (years), medication use (yes/no) and previous IBD-related surgery (yes/no)

### Interviews

Thirteen out of the fourteen interviewees reported to adhere to the Dutch physical activity guidelines of 150 min of moderate to high intensity exercise per week spread over several days. All in all, twelve out of the fourteen participants (86%) mentioned some kind of beneficial effect of physical activity. Regarding the role of physical activity in their disease, the difficulties experienced during physical activity were identified as one theme, whereas effects on disease activity, fatigue and quality of life were identified as three other themes. These four themes will be discussed in more detail below. Numbers do not always add up to fourteen, since interviewees could experience positive, negative or no effect of physical activity on disease activity, fatigue and quality of life, or sometimes they did not have an opinion about the effect.

#### Difficulties during physical activity

All interviewees reported to have experienced difficulties related to IBD that hindered their physical activity. Most commonly mentioned obstacles were frequent toilet use, pain and fatigue. Nine interviewees explained that the proximity of a toilet is important because they have unpredictable and urgent bowel movements.

“When I was a teen, the disease was such a burden I could not exercise at all. I was very dependent on the presence of a toilet. Physical activity affects my bowel movements. When someone asked me: “Do you want to come over and play soccer?”, I could not join because I knew there were no toilets anywhere nearby. Things like that can really have an influence in times of active disease.” (Interviewee 10)

Seven interviewees also mentioned that bowel movements can be induced by physical activity. One interviewee said that she would always bring some toilet paper with her when she would go for a run just in case, although her disease was not very active. Pain was also mentioned as a reason to not be physically active. Three interviewees were afraid of exercise-induced abdominal pain, and others already suffered from muscle pain and did not want to aggravate this. Six interviewees reported that they often are too fatigued to exercise. They said they could feel so tired they just wanted to lay down. In this case they found it very hard to gather enough motivation to exercise, so they would not do any physical activity.

#### Effects of physical activity on disease activity

Eight interviewees mentioned that physical activity can have positive effects on their disease activity, while five interviewees mentioned the opposite. Most commonly mentioned positive effects were that physical activity can help to improve overall fitness and muscle strength, making the body more resilient. Two interviewees also said that this resilience would help the body to recover quicker and better from flare-ups.

“I think it can have a positive effect in the sense that my body is always strong and fit, and I recover more quickly when I have a flare-up. The past six months my disease was very active. The doctors and I think that me being very physically active has prevented me from a colectomy and a stoma. We could keep trying different things without my body giving in, because it was strong.” (Interviewee 2)

Four interviewees also mentioned positive effects of physical activity on mental health. The distraction by physical activity is very important, this would take their mind off the pain they experience. Another interviewee mentioned that physical activity helps him to get a better mindset to help him cope with the disease. He mentioned that mind–body exercises are very important for him and help him to be aware of his body.

Negative effects were that physical activity, especially when including movements of the abdomen, can induce abdominal pain and bowel movement. Sit-ups, bending over, and jogging were mentioned as being too painful as the abdomen would move too much. Exercise-induced bowel movements were also mentioned as a negative effect. One interviewee mentioned she had to go to the toilet immediately when she tried to exercise, making physical activity very uncomfortable.

“When I start exercising, I have to go to the toilet. I get cramps and need to sit on the toilet for half an hour. Afterwards, I am all sweaty and I do not feel like exercising anymore.” (Interviewee 4)

#### Effects of physical activity on fatigue

Four interviewees were convinced physical activity could reduce fatigue. They mentioned it is harder to start exercising when they are fatigued, but they always feel more energetic after exercise than before.

“Sometimes when I come home from work, I feel like I cannot go any further. I am so extremely fatigued, something I do not recognise from before the diagnosis. Then I think, I have to train, and if I just do it, I always feel better afterwards. So, I think it is definitely good, even if you exercise on a lower level or speed. It requires a lot of discipline though. I totally understand that people with this disease do not feel like exercising anymore. It requires a lot of effort, it hurts, and you are tired. But I know it is good for me and if I do it, I feel better afterwards.” (Interviewee 2)

One interviewee said that when he is too tired, he would not exercise which only worsens his fatigue. He called it a vicious cycle.

#### Effects of physical activity on quality of life

Twelve interviewees noticed positive effects of physical activity on their quality of life. Physical activity leads to the feeling of being in shape, that they are healthier and can handle more physical setbacks. When interviewees felt physically fit, this also had a positive effect on their mental health, and it reduced stress levels. The social aspect of physical activity was also mentioned to be important for quality of life. Two interviewees said that they enjoyed physical activity because of the friends they cycle with, or the cup of coffee after a workout in the gym. Lastly, one interviewee mentioned that physical activity can help to recover quicker from a flare-up and thereby can improve her quality of life dramatically, from a score of 1 during severely active disease, to an 8 during remission. All twelve interviewees that noticed positive effects of physical activity on their quality of life indicated that when they are not able to exercise because of their disease activity, their quality of life is lower.

“When I can be more physically active, this would definitely have a positive effect. It would make me feel stronger, more in shape. You walk a bit more upright and you dare to show that you are there.” (Interviewee 7)

## Discussion

In this study, the level of physical activity was inversely associated with disease activity in participants with Crohn’s disease. This association was not found in participants with ulcerative colitis. Crohn’s disease participants who are less physically active seem to have a higher disease activity. Whether this association is causal remained unclear from the survey. However, interviews to gain insight in the nature of this association showed that most interviewees (86%) experience some kind of beneficial effect of physical activity. They reported that physical activity is very important to improve general fitness, quality of life, and self-image. However, in periods of active disease they found it is hard to find the motivation and perseverance to be physically active, since there are many physical barriers limiting physical activity. The proximity of a toilet was mentioned as a necessity for being able to exercise, and pain and fatigue were mentioned as obstructing factors.

Our data confirm that disease activity is associated with physical activity. A comparable result was found in a prospective study of 1857 IBD patients in remission. Patients with higher physical activity levels, measured by the Godin leisure-time activity index, were less likely to develop active disease at 6 months [22]. We showed that physical activity has the same association with disease activity in a more diverse patient group of CD patients, not only patients in remission, as we used the same questionnaires and cut-off points for disease activity. When comparing the physical activity rates of participants in our study to those in other studies, we found that physical activity rates vary widely between IBD cohorts, which may be explained by the heterogeneity of physical activity quantification and risk of selection bias [1, 9, 10, 23, 24]. Only one study used total physical activity scores and minutes per week to classify physical activity [24], just as in our study, whereas others only reported the times and degree of physical activity per week. The association found in CD, and the lack of an association in UC, is in line with previous studies: significant results are more commonly found in CD [22]. For each type of IBD a different disease activity questionnaire had to be used, which may induce bias. However, we found a similar distribution of disease activity groups in CD and UC, which makes it unlikely that the different questionnaires account for the difference in associations between CD and UC. The difference in associations might be explained by defective autophagy, which is only present in Crohn’s disease. It has been well established that exercise can induce autophagy, thereby it might exert a positive effect on disease activity [25, 26].

When looking into the nature of the association between physical activity and disease activity, interviewees reported that IBD-related complaints limit their physical activity for various reasons. Most commonly mentioned barriers were fatigue, exercise-induced pain, irregular and unpredictable bowel movements, and no proximity of a toilet; barriers that were also mentioned in previous studies [9, 24]. Several interviewees also mentioned immediate effects of physical activity on their symptoms. Inducement of bowel movement was mentioned the most, which could also result in cramps and abdominal pain. Similar exercise-induced symptoms were described in other studies, although these studies specifically focussed on high-intensity exercise [9, 27]. In the current study, not all interviewees defined the intensity of the exercise that caused symptom exacerbation but rather the mode of exercise. For example, running was mentioned to aggravate symptoms, whereas swimming was mentioned as an exercise mode that patients could endure for a long time without symptom aggravation. However, based on the MET scores, both are qualified as vigorous intensity exercise [20]. This indicates that not only the intensity of physical activity might influence symptoms, but the mode of exercise plays a crucial role. This is supported by, for example, runner’s diarrhoea, which is a common phenomenon in the general population, and this type of diarrhoea can also be present in IBD patients [28].

Besides immediate aggravation of symptoms, interviewees also mentioned alleviation of symptoms as a result of physical activity. Alleviation of fatigue was mentioned as an immediate effect. Interviewees often felt fatigued, but at the same time energized after physical activity. This finding is in line with a review supplemented with a case series which aimed to explore issues that clinicians may need to consider when giving exercise advice to IBD patients [7]. This study also reported that physical activity improved the mood of IBD patients. Mood improvement was also mentioned in the current study, as were long-term positive effects for example improved overall fitness, resilience, and positive effects on mental health. All these long-term effects were reported to have a beneficial effect on the quality of life of the interviewees, which is in line with existing literature [29, 30]. Interviewees mentioned that their quality of life could be improved by physical activity, but only when disease activity was low enough. Although not mentioned by the interviewees in this study, literature suggests that stress could aggravate disease activity and high stress levels can lead to a flare-up [5, 31, 32]. In line with previous studies, interviewees in the current study suggested that physical activity could reduce stress levels [33]. This could potentially mean that physical activity could reduce disease activity and the frequency of flare-ups by lowering stress levels and improving quality of life. However, more research on this topic is needed to confirm this hypothesis.

Strengths of this study include the combination of an online survey with interviews, with a large number of participants in the survey part, which enabled us to perform analyses for CD and UC separately, and the use of validated questionnaires to determine disease activity and physical activity. However, some limitations should be mentioned. Since the survey was cross-sectional any association could be due to reverse causality. In our case, it is probable that patients with more severe disease are less likely to be physically active, which was supported by the interviews, so we cannot conclude that lack of physical activity leads to higher disease activity. Next to this, our sample mainly included participants in remission. The power to find associations would have increased with an equal number of participants in each disease activity category. Another drawback is that we could not verify participants’ physical activity compliance. However, compliance applies to all participants at all times, and this systematic bias would not affect the association. An advantage of interviews is that they allowed us to discuss effects of physical activity during every disease stage, in contrast to the surveys in which only current disease activity was discussed. Another limitation of the survey is that we based disease activity on a questionnaire instead of an objective marker. However, previous studies validated the sCDAI and P-SCCAI and concluded that both are reliable and feasible for disease activity measurement [16, 17]. Final limitation of the survey is the self-reported data on medication and previous IBD-related surgery which could not be verified with medical records. A limitation of the interviews is that the intercoder reliability is small because the interviews were coded by one researcher only. However, all interviews were also performed by one researcher, which increases the comparability of the interviews, and regular discussion of the analysis within the research team acted as a quality control measure. Finally, separate analysis of the interviews for CD and UC would have been better to support the survey findings, but this was not possible since data saturation was not reached in these separate groups. However, our interviewees form a representative group of IBD patients.

## Conclusions

In conclusion, we found an inverse association between physical activity and disease activity in Crohn’s disease, but not in ulcerative colitis. No causal relationship has been proven with this association, but interviews with IBD patients suggest that it is prudent to stimulate IBD patients to be more physically active, as they generally experience beneficial effects from physical activity. When disease activity does not hinder IBD patients to be physically active, they experience an improved quality of life, reduced stress levels, less fatigue and a general feeling of fitness. Intervention studies are needed to further investigate the effect of lifestyle factors like physical activity on the course of disease.

## Supplementary Information

**Additional file 1.** Interview guide.

**Additional file 2.** Supplementary table 1 (Characteristics of the interview study population consisting of 7 CD and 7 UC participants).

## Data Availability

All data generated and/or analysed during the current study are available from the corresponding author on reasonable request.

## Abbreviations

ANOVA: Analysis of variance
BMI: Body mass index
CD: Crohn’s disease
CI: Confidence interval;
IBD: Inflammatory bowel disease
IQR: Interquartile range
MET: Metabolic equivalent of task
P-SCCAI: Patient Simple Clinical Colitis Activity Index
sCDAI: Short Crohn’s Disease Activity Index
SD: Standard deviation
SQUASH: Short Questionnaire to Assess Health enhancing physical activity
UC: Ulcerative colitis

## References

[1] Engels M, Cross RK, Long MD (2018). Exercise in patients with inflammatory bowel diseases: current perspectives. Clin Exp Gastroenterol.

[2] Packer N, Hoffman-Goetz L, Ward G (2010). Does physical activity affect quality of life, disease symptoms and immune measures in patients with inflammatory bowel disease? A systematic review. J Sports Med Phys Fitness.

[3] Monda V, Villano I, Messina A (2017). Exercise modifies the gut microbiota with positive health effects. Oxid Med Cell Longev.

[4] Ball D (2015). Metabolic and endocrine response to exercise: sympathoadrenal integration with skeletal muscle. J Endocrinol.

[5] Bitton A, Sewitch MJ, Peppercorn MA (2003). Psychosocial determinants of relapse in ulcerative colitis: a longitudinal study. Am J Gastroenterol.

[6] Vogelaar L, van’t Spijker A, Timman R (2014). Fatigue management in patients with IBD: a randomised controlled trial. Gut.

[7] Nathan I, Norton C, Czuber-Dochan W, Forbes A (2013). Exercise in individuals with inflammatory bowel disease. Gastroenterol Nurs.

[8] Gatt K, Schembri J, Katsanos KH (2019). Inflammatory bowel disease [IBD] and physical activity: a study on the impact of diagnosis on the level of exercise amongst patients with IBD. J Crohns Colitis.

[9] DeFilippis EM, Tabani S, Warren RU (2016). Exercise and self-reported limitations in patients with inflammatory bowel disease. Dig Dis Sci.

[10] Chan D, Robbins H, Rogers S (2014). Inflammatory bowel disease and exercise: results of a Crohn's and Colitis UK survey. Frontline Gastroenterol.

[11] Simrén M, Axelsson J, Gillberg R (2002). Quality of life in inflammatory bowel disease in remission: the impact of IBS-like symptoms and associated psychological factors. Am J Gastroenterol.

[12] Bernstein CN (2015). Treatment of IBD: where we are and where we are going. Am J Gastroenterol.

[13] Lamers CR, de Roos NM, Witteman BJM (2020). The association between inflammatory potential of diet and disease activity: results from a cross-sectional study in patients with inflammatory bowel disease. BMC Gastroenterol.

[14] Mikocka-Walus AA, Gordon AL, Stewart BJ, Andrews JM (2012). A magic pill? A qualitative analysis of patients' views on the role of antidepressant therapy in inflammatory bowel disease (IBD). BMC Gastroenterol.

[15] Hall NJ, Rubin GP, Dougall A (2005). The fight for 'health-related normality': a qualitative study of the experiences of individuals living with established inflammatory bowel disease (IBD). J Health Psychol.

[16] Thia K, Faubion WA, Loftus EV (2011). Short CDAI: development and validation of a shortened and simplified Crohn's disease activity index. Inflamm Bowel Dis.

[17] Bennebroek Evertsz F, Nieuwkerk PT, Stokkers PC (2013). The patient simple clinical colitis activity index (P-SCCAI) can detect ulcerative colitis (UC) disease activity in remission: a comparison of the P-SCCAI with clinician-based SCCAI and biological markers. J Crohns Colitis.

[18] Walsh AJ, Ghosh A, Brain AO (2014). Comparing disease activity indices in ulcerative colitis. J Crohns Colitis.

[19] Wendel-Vos GC, Schuit AJ, Saris WH, Kromhout D (2003). Reproducibility and relative validity of the short questionnaire to assess health-enhancing physical activity. J Clin Epidemiol.

[20] Ainsworth BE, Haskell WL, Herrmann SD (2011). 2011 Compendium of physical activities: a second update of codes and MET values. Med Sci Sports Exerc.

[21] Barriball KL, While A (1994). Collecting data using a semi-structured interview: a discussion paper. J Adv Nurs.

[22] Jones PD, Kappelman MD, Martin CF (2015). Exercise decreases risk of future active disease in patients with inflammatory bowel disease in remission. Inflamm Bowel Dis.

[23] D'Inca R, Garribba AT, Vettorato MG (2007). Use of alternative and complementary therapies by inflammatory bowel disease patients in an Italian tertiary referral centre. Dig Liver Dis.

[24] Tew GA, Jones K, Mikocka-Walus A (2016). Physical activity habits, limitations, and predictors in people with inflammatory bowel disease: a large cross-sectional online survey. Inflamm Bowel Dis.

[25] Wang Q, Xu K-Q, Qin X-R (2016). Association between physical activity and inflammatory bowel disease risk: a meta-analysis. Dig Liver Dis.

[26] Nys K, Agostinis P, Vermeire S (2013). Autophagy: a new target or an old strategy for the treatment of Crohn's disease?. Nat Rev Gastroenterol Hepatol.

[27] Bilski J, Brzozowski B, Mazur-Bialy A (2014). The role of physical exercise in inflammatory bowel disease. Biomed Res Int.

[28] de Oliveira EP (2017). Runner's diarrhea: what is it, what causes it, and how can it be prevented?. Curr Opin Gastroenterol.

[29] Ng V, Millard W, Lebrun C, Howard J (2007). Low-intensity exercise improves quality of life in patients with Crohn's disease. Clin J Sport Med.

[30] D'Incà R, Varnier M, Mestriner C (1999). Effect of moderate exercise on Crohn's disease patients in remission. Ital J Gastroenterol Hepatol.

[31] Mawdsley JE, Rampton DS (2005). Psychological stress in IBD: new insights into pathogenic and therapeutic implications. Gut.

[32] Bernstein CN, Singh S, Graff LA (2010). A prospective population-based study of triggers of symptomatic flares in IBD. Am J Gastroenterol.

[33] Hegberg NJ, Tone EB (2015). Physical activity and stress resilience: Considering those at-risk for developing mental health problems. Ment Health Phys Act.

