# Supplementary material for: Patient experiences with the role of physical activity in inflammatory bowel disease: results from a survey and interviews
Source: BMC Gastroenterol. 2021 Apr 14;21:172. doi: 10.1186/s12876-021-01739-z (PMC8046271; doi:10.1186/s12876-021-01739-z)
Supplement: Supplementary file 1 — Additional file 1. Interview guide. [file 12876_2021_1739_MOESM1_ESM.docx]

**Interview guide**

**Introduction**This interview is part of a study that investigates the influence of physical activity on complaints related to Crohn's disease or ulcerative colitis. To get a good and complete picture of what physical activity does to people with one of these inflammatory bowel diseases, it is important to hear the experiences of these people. The interview will take about half an hour. During this time we will discuss a number of topics; IBD and experienced complaints, physical activity, the link between IBD and physical activity and lastly the relation between IBD, quality of life, fatigue and physical activity. The interview will be recorded with your approval, for the sole purpose of transcribing the interview. The recordings will not be shared with people outside the research team and will be deleted directly after transcribing.

If you have any questions or comments, you can always ask or report them during the interview.

*(Fill in informed consent form)*

*(Start voice recorder)*

To begin with, I have a few questions to get a sense of the population I am interviewing

- What is your age?
- What is your highest level of education?

**IBD and experienced complaints**

- Do you have Crohn's disease or ulcerative colitis?
- Do you currently experience CD/UC related complaints?
- How do you notice IBD in your daily life?
  - What causes this?

**Physical activity in general**

- How physically active are you on an average day?
- Do you meet the advised level of physical activity of 30 minutes moderately intensive physical activity per day for at least 5 days a week? (Examples could be cycling to work or walking briskly)
- What is your profession?
  - Are you physically active at work?
  - How many days a week do you work?
- Do you perform many household activities and chores in and/or around the house?
- If you exercise, what kind of sports do you do?
  - Why?

**Link between IBD and physical activity**

- Do you feel that physical activity affects your IBD related disease burden?
  - If so: What is specifically affected?
  - Do you have a possible explanation for this?
- Are there things that prevent you from being physically active?
  - Does IBD play a role in this?
- Do you feel that physical activity affects your disease activity or vice versa; that your disease activity affects the degree to which you are physically active?

**Quality of life and fatigue**

- From one to ten, how would you rate your quality of life right now?
  - Which factors do you take into account when determining this rating?
  - What is the role of IBD in this rating?
  - Do you think physical activity plays a role in this rating?
- Do you suffer from fatigue?
  - If so: Do you feel that physical activity can affect fatigue?
  - If so: How does this work for you?
- Would you like to clarify or add something else?

**Closing**

This is the end of this interview. Is there anything you would like to explain further because you think I might be able to misinterpret it? Do you have any other questions? Or other things you would like to say?
