# Supplementary material for: Patient experiences with the role of physical activity in inflammatory bowel disease: results from a survey and interviews
Source: BMC Gastroenterol. 2021 Apr 14;21:172. doi: 10.1186/s12876-021-01739-z (PMC8046271; doi:10.1186/s12876-021-01739-z)
Supplement: Supplementary file 2 — Additional file 2. Supplementary table 1 (Characteristics of the interview study population consisting of 7 CD and 7 UC participants). [file 12876_2021_1739_MOESM2_ESM.docx]

| ***Supplementary Table 1.*** *Characteristics of the interview study population consisting of 7 CD and 7 UC participants* | | | | | |
| --- | --- | --- | --- | --- | --- |
|  | **Type of IBD**  CD / UC | **Gender**  male / female | **Age**  years | **BMI**  kg/m^2^ | **Education^#^**  low / middle / high |
| Interviewee 1 | UC | female | 63 | 27,1 | high |
| Interviewee 2 | UC | female | 35 | 20,0 | high |
| Interviewee 3 | UC | female | 78 | 26,5 | middle |
| Interviewee 4 | CD | female | 26 | 18,0 | high |
| Interviewee 5 | CD | male | 64 | 21,4 | low |
| Interviewee 6 | CD | female | 25 | 34,4 | middle |
| Interviewee 7 | CD | male | 50 | 21,6 | high |
| Interviewee 8 | UC | male | 70 | 46,7 | low |
| Interviewee 9 | CD | female | 71 | 23,0 | low |
| Interviewee 10 | UC | male | 29 | 26,1 | middle |
| Interviewee 11 | CD | male | 74 | 27,8 | high |
| Interviewee 12 | UC | male | 63 | 25,7 | middle |
| Interviewee 13 | CD | female | 56 | 25,7 | low |
| Interviewee 14 | UC | male | 58 | 23,4 | middle |
| Abbreviations: CD: Crohn’s disease, UC: ulcerative colitis, BMI: body mass index  ^#^ Education level: no education, primary or lower vocational education and lower general secondary education (low); secondary vocational education and higher general secondary education (middle); higher vocational education and university (high). | | | | | |
